# Willingness to Be Vaccinated against COVID-19 in Spain before the Start of Vaccination: A Cross-Sectional Study

**DOI:** 10.3390/ijerph18105272

**Published:** 2021-05-15

**Authors:** Noelia Rodríguez-Blanco, Sergio Montero-Navarro, José M. Botella-Rico, Antonio J. Felipe-Gómez, Jesús Sánchez-Más, José Tuells

**Affiliations:** 1 Department of Obstetrics and Gynaecology, Marina Baixa University Hospital, Av. Alcalde En Jaume Botella Mayor, 7, 03570 Villajoyosa, Spain; 2 Biomedical Sciences Department, Health Sciences Faculty, CEU-Cardenal Herrera University, CEU Universities, Plaza Reyes Católicos, 19, 03204 Elche, Spain; antonio.felipe@alumnos.uchceu.es (A.J.F.-G.); jesus.sanchez@uchceu.es (J.S.-M.); 3 Physical Therapy Department, Health Sciences Faculty, CEU-Cardenal Herrera University, CEU Universities, Plaza Reyes Católicos, 19, 03204 Elche, Spain; sergio.montero@uchceu.es (S.M.-N.); jmbotella@uchceu.es (J.M.B.-R.); 4 Department of Community Nursing, Preventive Medicine and Public Health and History of Science, University of Alicante, San Vicente del Raspeig, 03690 Alicante, Spain; tuells@ua.es

**Keywords:** vaccines, COVID-19, SARS-CoV-2, vaccine acceptance, vaccine hesitancy, vaccination campaign, immunization program

## Abstract

Vaccine hesitancy has increased in the past few years, influenced by the socio-cultural differences, political populism, or concerns related to the effectiveness and safety of some vaccines, resulting a feeling of distrust. This feeling can become a barrier against the achievement of the immunity necessary to stop the expansion of COVID-19. The aim of this study was to evaluate the acceptance of the vaccine against COVID-19 in Spain, as well as to identify the factors that have an influence on the concerns and attitudes of people against accepting the vaccine in the months prior to the start of vaccination on December 2020. An online questionnaire was created to obtain information about (1) sociodemographic characteristics; (2) concerns and sources of information about vaccines; and (3) attitudes about vaccination and state of health. A multivariate logistic regression was performed to identify the influencing factors. Of the 2501 participants, 1207 (48.3%) would accept the COVID-19 vaccine, 623 (24.9%) were hesitant, and 671 (26.8%) would reject it. The logistic regression showed that being male, older than 60, married, retired, with a high level of education, or with a leftist political inclination, could increase the probability of accepting the COVID-19 vaccine. Disinformation and the lack of political consensus were the main sources of distrust. The patients with hypertension, immunodepression, hypercholesterolemia, or respiratory disease, or were overweight, showed a greater acceptance to the vaccine, while those with cancer took the longest to accept it. A low acceptance of the vaccine against COVID-19 was observed among the Spanish population in the phase prior to its availability, and the main fears of the population were identified. It is necessary to offer correct and transparent information about these vaccines to reduce the concerns and increase the trust of the population, to thereby guarantee the success of the vaccination campaigns.

## 1. Introduction

Spain is one of the countries which has been greatly affected by the SARS-CoV-2 virus, with more than 74,064 deaths and more than 3,241,345 confirmed cases until now [1]. Vaccination against COVID-19 is the best strategy for mitigating the expansion of the disease; thus, achieving a reasonable herd immunity is necessary. In Spain, the vaccination campaign began on 27th December 2020, in unison with the 27 EU countries. Although these date were symbolic, vaccination begun immediately with a scheme which prioritized certain population groups: individuals living in elderly homes, the workers in these centers, frontline health workers, and dependents [2]. However, there is a latent risk that feelings of rejection could emerge that are associated with the secondary effects from the vaccine, a low literacy related to health, or a low perception of risk of becoming infected [3,4]. Vaccine hesitancy has steadily increased since 2014 [5], which could become a severe problem in the fight against COVID-19, as was already observed during the 2009 H1N1 Influenza A Pandemic [6,7,8]. At present, socio-cultural differences, the increase in political populism, and concerns about the effectiveness or safety of these new vaccines have been observed, as well as an excess of false news in the media and social networks that have created mistrust in the population [3,9,10,11]. 

As recommended by the WHO, it is very important to periodically evaluate the attitude and pre-disposition of the population associated to the vaccine against COVID-19, to implement measures that could guide the vaccination campaign and increase the acceptance and demand for vaccination [12]. It has been said that the end of the pandemic could be glimpsed when 70% of the world’s population, approximately 5.6 billion people, is immunized, and to achieve this objective, solid rates of vaccination in every country are needed [13,14]; however, this has been questioned recently [15].

The studies published prior to the start of the vaccination campaign have shown different vaccine hesitancy results depending on the date consulted, from 9–12% in China, 14–28% in the UK, 22.4% in Spain, 25–33% in the USA, to 41% and 45% in France and Russia, respectively [16,17,18,19,20]. It is known that the acceptance of a vaccine by the same population group could vary as the start of vaccination nears, and that it greatly depends on the communication strategy utilized by the institutions. In this sense, the Spanish Government insisted on the importance of a good communication strategy and equal access to it before the arrival of the vaccine to Spain [21]. 

The aim of the study was to find out the percentage of acceptance of the vaccines for the prevention of the COVID-19 disease in the Spanish population just before the start of the vaccination campaign, as well as to identify the factors that have an influence on the concerns and attitudes of the people for accepting them.

## 2. Materials and Methods

### 2.1. Design, Population, and Sample

A cross-sectional descriptive study was conducted, starting on 26th November 2020, and ending on 26th December, coinciding with the day prior to the start of vaccination in Spain. The study used an electronic questionnaire and included all those older than 18 who resided in Spain, and who utilized social networks such as WhatsApp, Facebook, and Instagram, and smartphones. The Spanish population aged 18 or older, numbering 40,631,764 individuals, as estimated by the National Statistics Institute (INE) as of 1 July 2020, was used as the reference population [22]. The calculation of the sample size was 385, with a level of confidence of 95%, and a margin of error of 5% [23]. 

### 2.2. Data-Collection Tool

A questionnaire designed ad hoc, based on previous studies [24,25], was utilized as the data-collection instrument, and a pilot study was conducted with a group of 50 health sciences university students (25 men and 25 women), who were not taken into account for this analysis. This pilot study allowed us to collect the impressions of those polled, which were afterwards evaluated by a group of experts to redesign the questionnaire and guarantee the validity of the different items. The final questionnaire was composed of 23 questions, which included the following: (1) sociodemographic characteristics (age, sex, marital status, education, employment, economic status, political inclination, and religion); (2) concerns and sources of information about vaccines; and (3) attitudes towards vaccination and the current state of health. All the questions were close-ended. The voluntary consent, objectives of the study, the code of acceptance from the ethics committee, and the estimated length of time needed for completing the questionnaire were included on the questionnaire heading.

### 2.3. The Dependent Variable

The dependent variable was the acceptance of the COVID-19 vaccine. The individuals who answered “yes” to the question “When the Covid-19 vaccine is available, are you willing to receive it as soon as possible?” were classified into the acceptance group, and those who answered “no” were assigned to the rejection group, while those who answered “I don’t know” were assigned to the hesitant group. A total of 2621 questionnaires were obtained, with a final sample of 2501 due to the exclusion of 120 questionnaires in which the first question had not been answered.

### 2.4. Methods of Analysis

The mean ± standard deviation was utilized for the quantitative variables, and frequency tables for the qualitative data. The Chi-square test was utilized to investigate the relationships between the categorical variables. The factors associated to the willingness to receive the vaccine were identified through the use a logistic regression analysis. A multivariate logistic regression was performed between the three groups (acceptance group vs. rejection group or hesitant group) to identify the factors which had an influence of vaccine acceptance, with the odds ratio (OR) probability and a confidence interval (CI) of 95% calculated. The likelihood was calculated with Wald Chi-square test, and the goodness-of-fit was tested by Pearson’s test. The statistical analysis was performed with the IBM SPSS Statistics para Windows, version 24.0.

### 2.5. Ethical Considerations

The study was conducted in accordance to the principles from the Declaration of Helsinki on human clinical trials, and the research proposal was approved in November 2020, by the Ethics Committee from the CEU Cardenal-Herrera University (CEI20/094).

## 3. Results

### 3.1. Population Description

The detailed characteristics of the participants, and the statistical analysis of the explanatory variables of the decision to accept the vaccine are found in Table 1. Of the 2501 participants who answered the main study question “When the Covid-19 vaccine is available, are you willing to receive it as soon as possible?” 48.3% (1207) answered affirmatively. Moreover, 51.7% of the participants rejected (671) or were hesitant (623) about accepting the COVID-19 vaccine. The mean age of the participants was 40.2 ± 13.6 (18–97), and 71.8% were women. (Table 1). 

### 3.2. Variables with Influence on the Decision

As for the factors that had an influence on the decision to accept the vaccine, the women had the most negative opinions (not being vaccinated/indecisive) as compared to the men (*p* < 0.001). The participants with partners were more willing to be vaccinated than those who were single, divorced, or widowed.

As for their age, it was observed that, as aged increased, so did the acceptance to become vaccinated, so that the group with the greatest acceptance rate was those older than 60 years old (59.2%, *p* = 0.001 vs. 18–29-years-old group). These data are in agreement with the employment, with those who were retired being the ones who showed the greatest acceptance of the vaccine (*p* = 0.013), while the students showed the greatest rejection towards it (*p* = 0.039). Within the employed group, an influence was not observed of the professional field when accepting the vaccine.

As for education, the university students had the greatest vaccine-acceptance rate, while the other groups with less education showed the greatest hesitancy against accepting the vaccine. Economic status was also an influencing factor when making the decision to accept the vaccine, with the middle class showing the greatest acceptance, and the lower class showing the greatest rejection (*p* = 0.004).

Religion was not a conditioning factor for the decision to accept or reject the vaccine; however, the non-religious were less hesitant than those who were Christian (*p* = 0.017). On the contrary, the political inclination was a determining factor, with the left-leaning participants being the ones who showed the greatest acceptance towards the vaccine, and those who were right-leaning showing the greatest rejection and hesitancy (*p* < 0.001).

### 3.3. Acceptance, Rejection, or Hesitancy of the Vaccination through Time

**Figure 1:**
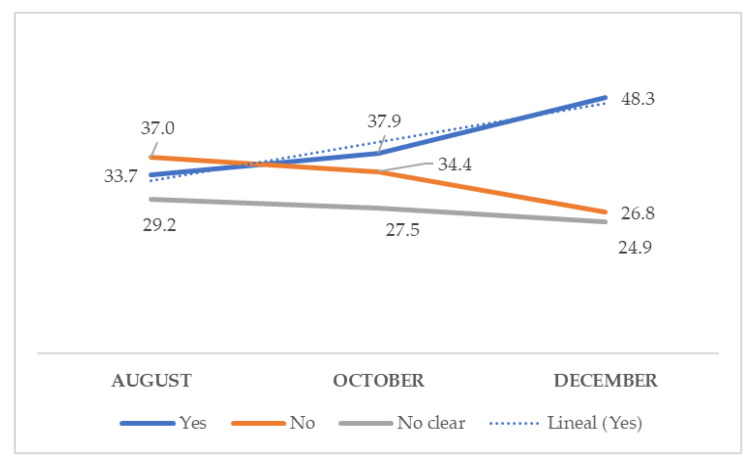
Evolution of vaccine acceptance through time.

Figure 1 shows the percentages of acceptance, rejection, or hesitancy of the vaccine in the different periods prior to the start of the vaccination campaign. Aside from asking the participants if they would be vaccinated in December, when the vaccination campaign began in Spain, they were also asked if they would have been vaccinated if the vaccine had been available in the months of August or October. A progressive increase was observed in the percentage of individuals who would accept the COVID-19 vaccine as the vaccination acquisition time neared, and the resulting decrease of rejection or hesitancy.

### 3.4. Concerns and Sources of Information about the Vaccine

The “lack of information about secondary effects” and the “speed with which it was created” were the most common reasons for hesitancy or rejection of the vaccine. The variety of information related to the vaccine and the diversity of social agents responsible for communicating the information were also some of the factors that were more associated with the high percentage of rejection or hesitancy among the population (Table 2). 

The press, the communication media, and the social networks were the main sources of information consulted by the population prior to the start of vaccination. To a lesser degree, the population consulted other sources that were more specialized, such as the webpages of organizations and associations related to health, or scientific bibliographic databases. The social agents trusted by the population were the health workers (91.8%), and it is through their recommendation that the percentage of vaccine acceptance (62.5%) could be increased.

### 3.5. Multivariate Logistic Regression Analyses Showing Attitudes of the Participants as Factors Associated with Acceptance of a COVID-19 Vaccine in Spain

The beliefs of the population associated to vaccines is a conditioning factor for the acceptance or rejection of COVID-19. The individuals whose children have completed the vaccination schedule, or those who habitually vaccinate against the flu every year, were the ones who had the greatest willingness to accept the vaccine against COVID-19 (*p* < 0.001) (Table 3). Likewise, the participants who believed that the vaccines could cause the disease it tries to prevent showed the greatest rejection and hesitation about the vaccine (*p* < 0.001), while those with the opinion that without the vaccines there would be a greater incidence of infectious diseases showed the greatest acceptance of the COVID-19 vaccine (*p* < 0.001)

Table 4 shows the results from the question of if the state of health had an influence on the decision of accepting the COVID-19 vaccine. The results showed a higher acceptance from the participants who suffered from immunodepression (*p* = 0.014), overweightness (*p* = 0.002), hypertension (*p* = 0.006), respiratory disease (*p* = 0.001), or hypercholesterolemia (*p* = 0.032). However, cancer patients showed a significant hesitancy (*p* = 0.003). 

## 4. Discussion

### 4.1. Population: Vaccine Acceptance

At the end of 2020, the massive vaccination campaigns begun in Spain, prioritizing the most vulnerable individuals after the approval of two vaccines (Comirnaty by Pfizer-BionTech, and COVID-19 Vaccine by Moderna) by the European Medicines Agency [26]. The common objective of every vaccination campaign is to create a high degree of confidence among the population, which will be key for achieving the high rates of vaccination coverage needed for reaching herd immunity [21,27,28]. 

The present study demonstrated the low acceptance of the vaccine against COVID-19 among the Spanish population (48.3%) at the end of 2020, just before the start of the vaccination campaign. There are only two previous studies that had analyzed the percent of acceptance of the new COVID-19 vaccine in the Spanish population in the period prior to the start of the vaccination campaign [16,20].

In the study by Lazarus et al., the population was asked about acceptance in June 2020, with 74.33% stating that they would accept it when it became available [16], while the study by Eguia et al. asked individuals the same question between September and November 2020, with a 77.5% rate of acceptance observed [20]. These data are higher than the results from our study, where the acceptance in August was only 33.7%, but then progressively increased to 48.3% in December. The study by Lazarus et al. did not show the sociodemographic characteristics of the population, while the study by Eguia et al. only showed data referring to age, sex, or profession, which did not allow us to determine if the differences found were due to possible population biases.

In any case, in both studies, the participation was lower than 800 individuals, which is inferior to the sample utilized in our study (*n* = 2501).

Our results showed a higher rejection than other studies conducted in March in France, and in May in the USA, where only 26% and 20%, respectively, would reject the vaccine [29,30], which makes us think about an increase in acceptance at the start of the vaccination campaign. However, the hesitancy could even increase after the introduction of the vaccine, as observed in France with the H1N1 pandemic, when only 10% received the vaccine as compared to the 27% who had the intention of being vaccinated [6,7,8]. To avoid this situation, the suggestion was made to periodically analyze, during the vaccination campaign, the profile of individuals who have doubts about the effectiveness of vaccines in Spain, where vaccination coverage is the highest in Europe in the children population but decreases in the adult stages, to implement measures that guarantee a high percentage of acceptance of the vaccine [3]. 

### 4.2. Acceptance, Rejection or Vaccine Hesitancy through Time

The main statistically significant independent variables associated with vaccine acceptance were being male, older, living with a partner, and being retired. These data were in agreement with those from a European poll, where the men older than 55 years old were more willing to vaccinate [31]. However, not all the studies coincided with the age of greatest acceptance; thus, as opposed to our study, the groups which showed the greatest rejection were those older than 75 years old, despite being a very vulnerable age group [30]. The type of employment did not have an influence on vaccine rejection; however, a lower level of education was associated with the rejection of vaccines, as previously described in other studies conducted in Syria and Australia [32,33,34]. There was evidence that political leanings played an important role on the attitude associated to vaccines. Thus, in France, those who had voted for an extreme left or extreme right candidate had a higher probability of rejecting the COVID-19 vaccine [19]. Our study confirmed the influence of the political leaning when accepting the vaccine; however, in Spain, the left-leaning voters were the ones who showed the greatest acceptance, and the right-leaning ones who showed the greatest rejection.

The growing anti-vaccine sentiment in the last few years is known [5]. The consequences of vaccine hesitancy are also reflected in that some parents ask for the delay of the vaccine dose, so that they shift away from the recommended vaccination calendar [35]. In this work, 24.9% hesitated being vaccinated, waiting for more data on the safety of the vaccine [17]. In this sense, our study showed that those who had children and complied with the vaccination calendar were more willing to be vaccinated. At present, vaccines for those younger than 16 are not available, so that the feeling of protection after immunization could be the reason behind the acceptance in this population group [36]. Being vaccinated against the flu in other seasons [37,38] and a completed children’s vaccination calendar of individuals with children were also variables that exerted a very favorable trend in vaccine acceptance.

### 4.3. Concerns and Sources of Vaccine-Related Information

The manifestation of “fear of the secondary effects” was repeated in this and other studies as the main cause for vaccine hesitation [4,20,39]. The high percentage of hesitation in the Spanish population could be partly explained by the rejection of the government’s management in 2020, as indicated by the low confidence in the politicians, the concern when facing a lack of political consensus, and the influence of the political inclination when accepting the vaccine, as shown in our study [30,40]. It is known that mistrust in political populism can promote the feeling against vaccination, creating confusion and provoking mistrust [10,33]. Thus, familiarity and trust on the messenger, the coherence of the messages, and the political management, are fundamental for succeeding against vaccine hesitancy [12,41]. In crisis situations, the interpersonal trust tends to increase, while the institutional trust tends to decrease, mainly because of the mistrust of the government’s recommendations [3,42]. Politicians, more than health professionals, are the public face of crisis management, a mistake that was demonstrated in France with the management of the H1N1 flu in 2009 [6,7,8]. The rupture of political unity and the doubts associated with the speed with which the vaccine was developed resulted in a low acceptance of the vaccine (only 10% of the population was vaccinated) [7,43].

### 4.4. A multivariate Logistic Regression Analysis Shows the Attitudes of the Participants as Factors Associated to COVID Vaccine Acceptance in Spain

Our results show that vaccine acceptance increases over time, as the period of vaccination nears in Spain, surely influenced by the increased information related to it, but the lack of political unity and consensus, together with the doubts of social agents with recognized prestige, could increase the doubts of the population. The recommendation and advice from a health professional are therefore key for achieving a high vaccination coverage in the vaccination calendar [13,17,34] and a high herd immunity [44]. In this work, the health experts were pointed out as the main agents in which the population would place its trust. As for the sources of information, the press and the radio were cited as the most utilized, along with the social networks, which represented a high percentage (25%). These last, more social sources of information could be linked to false news or non-contrasted statements that could derive into an increased hesitancy [39]. Due to this, scientists from the area of health could be ones who must play the information dissemination role for the population, to transmit the latest findings [45]. 

Another of the main concerns of the Spanish population was the speed with which the COVID-19 vaccine was created. Although the unprecedented situation we are currently living in demanded the acceleration in the development of the vaccine, conflicts of interests were put forward [46]. The lack of knowledge about the secondary effects of a new vaccine increases the level of mistrust, also thanks to contradictory information and “fake news” that reach the population through different means. In this sense, the communication media, the traditional ones, and the social networks, must significantly contribute to address these fears [9,43,47]. The WHO points out that transparency and personalized information on the evolution of the epidemiological risk in the community are key aspects for obtaining the trust of the population [27,28].

### 4.5. Study Limitations

The limitations of the study were that the results are strongly marked by a social context of constant changes in confinement policies in Spain and a great variability in the information offered to the population about the vaccines that were not yet available. Regarding the questionnaire, a non-validated new survey was applied, and the methodology used for its dissemination meant that all the data were self-reported by the participants, with a high level of education and a high participation of health professionals, which could result in response bias. However, the large sample obtained and its anonymity were some of its strengths.

### 4.6. Practical Implications

In the present work, as practical implications and future research, we evaluated the intention to become vaccinated. We obtained a gradual increase in the acceptability of the vaccines as their availability came closer, but this percentage could decrease with the final administration if adequate communication strategies with the population are not implemented, during a world distribution scenario in 2021 [48,49]. 

The vaccination strategies for 2021 should be adapted to a reality in which a society is concerned about the pandemic, where the determinants of vaccine hesitancy are complex and varying across time, place, and type of vaccine, by identifying the barriers in the current context [24,50]. 

## 5. Conclusions

In the Spanish population analyzed prior to the start of the vaccination campaign at the end of 2020, the percentage of acceptance of the new vaccines aimed at preventing the COVID-19 disease was low. Disinformation and the lack of political consensus are the main doubts of the Spanish population associated to the new vaccines against SARS-CoV-2 in an extraordinary scientific-health context.

In this study, the participating population had a low rate of acceptance of the vaccines destined to fight against COVID-19, caused by concerns about side effects, the speed with which it was created, and the thought that it may not be reliable. Communication with the population must be as personalized as possible, as we found the existence of age groups, levels of education, political ideology, beliefs about vaccines in general, and the state of health itself, which have an influence on vaccine acceptance or rejection.

There is, thus, a need for monitoring and planning of the vaccination campaign, which, in many countries, will be accelerated [47,51]. Trust in the institutions is fundamental to guarantee the levels of vaccination that lead to herd immunity.

## Figures and Tables

**Table 1:** Sociodemographic characteristics of the participants and multivariate logistic regression analyses showing factors associated with acceptance of a COVID-19 vaccine in Spain.

Variable (*n*)	Accept	Rejection			Hesitation		
	*n* (%)	*n* (%)	OR (95% CI)	*p*-Value	*n* (%)	OR (95% CI)	*p*-Value
Gender (2494)							
Male (R)	396 (55.9)	164 (23.2)	1	<0.001	148 (23.9)	1	0.001
Female	809 (45.3)	506 (28.3)	1.51 (1.22–1.87)		471 (26.4)	1.55 (1.25–1.94)	
Civil status (2494)							
Couple (R)	802 (51.5)	388 (24.9)	1	0.001	366 (23.5)	1	0.02
Single	316 (43.9)	217 (30.1)	1.42 (1.15–1.75)	0.043	187 (26.0)	1.30 (1.04–1.61)	0.011
Divorced	88 (41.5)	61 (28.8)	1.43 (1.01–2.03)		63 (29.7)	1.57 (1.11–2.22)	
Age group (2412)							
18–29 (R)	289 (45.5)	182 (28.7)	1	1	164 (25.8)	1	1
30–39	206 (44.9)	134 (29.2)	1.03 (0.78–1.38)	0.824	119 (25.9)	1.02(0.76–1.37)	0.906
40–49	368 (49.5)	200 (30.7)	0.86 (0.67–1.11)	0.254	176 (23.7)	0.84 (0.65–1.10)	0.202
50–59	178 (47.7)	99 (15.2)	0.88 (0.65–1.20)	0.429	96 (25.7)	0.95 (0.67–1.30)	0.750
>60	119 (59.2)	36 (17.9)	0.48 (0.32–0.73)	0.001	46 (7.7)	0.68 (0.46–1.00)	0.054
Employment (2461)							
Worker (R)	909 (50.2)	486 (26.8)	1	1	416 (23.0)	1	1
Unemployed	30 (39.5)	20 (26.3)	1.25 (0.70–2.22)	0.453	26 (34.2)	1.89 (1.11–3.24)	0.020
Retired	71 (55.5)	20 (15.6)	0.53 (0.32–0.88)	0.013	37 (28.9)	1.14 (0.75–1.72)	0.539
Student	186 (41.7)	131 (29.4)	1.32 (1.03–1.69)	0.03	129 (28.9)	1.51 (1.18–1.95)	0.001
Type of work (1811)							
Health (R)	363 (52.0)	179 (25.6)	1	1	156 (22.3)	1	1
Humanities	130 (508)	71 (27.7)	1.11 (0.79–1.56)	0.556	55 (51.5)	0.98 (0.68–1.42)	0.933
Social	161 (45.9)	106 (30.2)	1.34 (0.99–1.81)	0.062	84 (23.9)	1.20 (0.88–1.68)	0.240
Services	197 (49.0)	104 (25.9)	1.07 (0.80–1.44)	0.653	101 (25.1)	1.19 (0.88–1.62)	0.256
Others	58 (55.8)	26 (25.0)	0.91 (0.55–1.49)	0.706	20 (19.2)	0.80 (0.47–1.38)	0.426
Level of study (2483)							
University (R)	761 (51.7)	374 (25.4)	1	1	336 (22.8)	1	1
Vocational training	135 (38.2)	130 (36.8)	1.96 (1.50–2.57)	<0.001	88 (24.9)	1.48 (1.10–2.00)	0.010
A-level degree	156 (43.6)	94 (26.3)	1.23 (0.92–0.162)	0.160	108 (30.2)	1.57 (1.19–2.01)	0.001
High school	147 (48.8)	65 (21.6)	0.90 (0.66–1.23)	0.514	89 (29.6)	1.37 (1.02–1.84)	0.035
Economic status (2487)							
Medium (R)	802 (50.9)	383 (24.3)	1	1	392 (24.9)	1	1
Low	28 (38.9)	29 (40.3)	2.17 (1.27–3.70)	0.004	15 (20.8)	1.10 (0.58–2.01)	0.78
Low/Medium	211 (44.4)	137 (28.8)	1.36 (1.06–1.74)	0.015	127 (26.7)	1.23 (0.96–1.58)	0.10
Medium/High	159 (44.9)	112 (31.6)	1.47 (1.12–1.93)	0.005	83 (23.4)	1.07 (0.80–1.43)	0.66
High	5 (55.6)	4 (44.4)	1.68 (0.45–6.27)	0.444	0 (0)	-	
Religion (2456)							
Christian (R)	651 (46.8)	371 (26.7)	1	1	369 (26.5)	1	1
None	533 (51.0)	276 (26.4)	0.91 (0.75–1.10)	0.331	237 (22.7)	0.78 (0.64–0.96)	0.017
Others	6 (31.6)	6 (31.6)	1.76 (0.56–5.48)	0.333	7 (36.8)	2.058 (0.69–6.17)	0.198
Political inclination (2303)							
Right (R)	232 (39.4)	202 (34.3)	1	1	155 (26.3)	1	1
Center	389 (47.3)	210 (25.5)	0.62 (0.48–0.80)	<0.001	224 (27.2)	0.86 (0.66–1.12)	0.265
Left	534 (59.9)	171 (19.2)	0.37 (0.29–0.48)	<0.001	186 (20.9)	0.52 (0.40–0.68)	0.001

OR = is calculated for each answer as compared to all the others; *p*-value *=* calculated for the Chi-square test group.

**Table 2:** Concerns associated with the Covid-19 vaccine and sources of information consulted by participants.

What Do You Think May be the Main Problem(s) that Leads to Doubts against Becoming Vaccinated with Covid-19 when the Vaccine Becomes Available?	
Misinformation about the side effects that the vaccine may have	1757 (70.9)
How quickly its creation has taken place	1293 (52.2)
The variety of information from different media	693 (28.0)
That there are social agents of recognized prestige who doubt the vaccine	627 (25.3)
Lack of consensus among political leaders in developing vaccination policies	377 (15.2)
There is no factor that raises doubts	35 (1.4)
From what sources do you collect information regarding the Covid-19 vaccine?	
Written or digital press	1226 (49.5)
Television or radio	949 (38.3)
Social media	638 (25.8)
Official pages of health-related organizations and associations and medical bibliographic bases	499 (20.1)
Google-type search engines	458 (18.5)
No	9 (0.4)
Which of the following social agents do you trust the most?	
Health	2295 (91.8)
Spiritual leaders	35 (1.4)
Journalists	11 (0.4)
Political	10 (0.4)
Other	15 (0.6)
None at all	99 (4.0)
Not clear	14 (0.6)
No answer provided	22 (0.9)
If the social agent you have selected recommends that you get the COVID-19 vaccine when it is ready, will you do it?	
Yes	1555 (62.5)
No	329 (13.2)
Not clear	606 (24.3)

**Table 3:** Multivariate logistic regression analyses showing attitudes of the participants as factors associated with acceptance of a COVID-19 vaccine in Spain.

Variable (*n*)	Accept	Rejection			Hesitation		
	*n* (%)	*n* (%)	OR (95% CI)	*p*-Value	*n* (%)	OR (95% CI)	*p*-Value
In the case that you have children, will you receive the COVID-19 vaccine when it becomes available? (2324)							
No (R)	44 (6.1)	580 (79.8)	1		103 (14.2)	1	
Yes	846 (96.1)	11 (1.2)	0.001 (0.001–0.02)	<0.001	24 (2.7)	0.01 (0.01–0.02)	<0.001
Not clear	220 (30.7)	49 (6.8)	0.02 (0.01–0.03)	<0.001	447 (62.4)	0.87 (0.49–1.23)	0.475
Do you usually receive the vaccine against the flu? (2495)							
No (R)	529 (38.1)	501 (36.1)	1		359 (25.8)	1	
Yes	501 (63.2)	115 (14.5)	0.24 (0.19–0.31)	<0.001	177 (22.3)	0.52 (0.42–0.65)	<0.001
Occasionally	172 (55.0)	60 (19.2)	0.37 (0.27–0.51)	<0.001	81 (25.9)	0.70 (0.52–0.93)	0.016
Do you believe that, without vaccines, the population would suffer more diseases, such as measles, chickenpox, etc.? (2499)							
No (R)	26 (22.6)	77 (67.0)	1		12 (10.4)	1	
Yes	1158 (51.5)	530 (23.6)	0.16 (0.10–0.24)	<0.001	562 (25.0)	1.05 (0.53–2.10)	0.887
Not clear	19 (14.2)	70 (52.2)	1.24 (0.63–2.44)	0.526	45 (33.6)	5.13 (2.15–12.2)	<0.001
Do you believe that vaccines can cause more diseases that they intend to prevent? (2500)							
No (R)	906 (58.9)	307 (20.0)	1		325 (21.1)	1	
Yes	113 (26.3)	202 (47.1)	5.28 (4.05–6.87)	<0.001	114 (26.6)	2.81 (2.11–3.76)	<0.001
Not clear	186 (34.9)	168 (31.5)	2.67 (2.09–3.41)	<0.001	179 (33.6)	2.68 (2.11–3.42)	<0.001
In the case that you have children, do they have the vaccination calendar up to date? (2389)							
No (R)	6 (20.0)	20 (66.7)	1		4 (13.3)	1	
Yes	702 (49.9)	361 (25.5)	0.15 (0.06–0.39)	<0.001	354 (25.0)	0.76 (0.21–2.70)	0.667
Not clear	2 (18.2)	3 (27.3)	0.45 (0.06–3.35)	0.436	6 (54.5)	4.5 (0.59–34.6)	0.148
Not children	442 (47.5)	264 (28.4)	0.18 (0.71–0.45)	<0.001	225 (24.2)	0.76 (0.21–2.73)	0.678

OR = is calculated for each answer as compared to all the others; *p*-value *=* calculated for the Chi-square test group.

**Table 4:** Multivariate logistic regression analyses showing health status as factor associated with acceptance of a COVID-19 vaccine in Spain (*n* = 2457).

Variable	Accept	Rejection			Hesitation		
	*n* (%)	*n* (%)	OR (95% CI)	*p*-Value	*n* (%)	OR (95% CI)	*p*-Value
Cancer							
No	1189 (48.8)	651 (26.7)	0.46 (0.14–1.50)	0.196	594 (24.4)	0.21 (0.07–0.59)	0.003
Yes (R)	5 (21.7)	6 (26.1)	1		12 (52.2)	1	
Respiratory disease							
No	1121 (47.8)	627 (26.7)	0.46 (0.14–1.5)	0.456	597 (25.5)	4.35 (2.15–8.80)	0.001
Yes (R)	73 (65.2)	30 (26.8)	1		9 (8.0)	1	
Diabetes							
No	1156 (48.3)	643 (26.9)	1.51 (0.81–2.80)	0.193	591 (24.8)	1.62 (0.84–3.14)	0.146
Yes (R)	38 (59.4)	14 (21.9)	1		12 (18.8)	1	
Cardiac disease							
No	1170 (48.5)	647 (26.8)	1.33 (0.63–2.79)	0.456	593 (24.6)	0.94 (0.47–1.85)	0.936
Yes (R)	24 (51.1)	10 (21.3)	1		13 (27.7)	1	
Liver disease							
No	1187 (48.5)	655 (26.8)	1.93 (0.40–9.32)	0.413	606 (24.8)	-	-
Yes (R)	7 (77.8)	2 (22.2)	1		0 (0.0)	1	
Renal disease							
No	1190 (48.7)	654 26.8)	0.73 (0.16–3.28)	0.685	600 (24.5)	0.34 (0.09–1.20)	0.092
Yes (R)	4 (30.8)	3 (23.1)	1		6 (46.2)	1	
Hypercholesterolemia							
No	1122 (48.1)	629 (26.9)	1.44 (0.92–2.26)	0.109	584 (25.0)	1.70 (1.05–2.78)	0.032
Yes (R)	72 (59.0)	28 (23.0)	1		22 (18.0)	1	
Immunodepression							
No	1162 (48.3)	651 (27.0)	2.99 (1.24–7.18)	0.014	595 (24.7)	1.49 (0.75–2.98)	0.259
Yes (R)	32 (65.3)	6 (12.2)	1		11 (22.4)	1	
Overweightness							
No	995 (47.3)	583 (27.7)	1.58 (1.18–2.10)	0.002	524 (24.9)	1.28 (0.97–1.69)	0.084
Yes (R)	199 (56.1)	74 (20.8)	1		82 (23.1)	1	
Hypertension							
No	1081 (48.0)	619 (27.5)	1.70 (1.16–2.49)	0.006	553 (24.5)	1.09 (0.78–1.54)	0.619
Yes (R)	113 (55.4)	38 (18.6)	1		53 (26.0)	1	

OR = is calculated for each answer as compared to all the others; *p*-value *=* calculated for the Chi-square test group.

## Data Availability

The data presented in this study are available on reasonable request from the corresponding author. The data are not publicly available, due to ethical requirements.
